# Recurrent Laryngeal Nerve Identification in Thyroidectomy: Limitations of Anatomical Landmarks and the Gamboa-Hoil Principle

**DOI:** 10.7759/cureus.107506

**Published:** 2026-04-21

**Authors:** Sergio Isidro Gamboa-Hoil

**Affiliations:** 1 Surgical Oncology, Mexican Social Security Institute, Merida, MEX

**Keywords:** anatomical variability, gamboa-hoil principle, nerve injury, recurrent laryngeal nerve, thyroidectomy

## Abstract

Recurrent laryngeal nerve (RLN) injury remains one of the most clinically significant complications of thyroidectomy, with potential consequences for voice, airway protection, and overall quality of life. Despite advances in surgical technique, the reliable identification and preservation of the RLN remain challenging because of its anatomical variability. This narrative review analyzes the anatomical course of the RLN and its relationship with commonly used surgical landmarks, including Berry’s ligament (BL), the Zuckerkandl tubercle (ZT), the tracheoesophageal groove (TEG), and the inferior thyroid artery (ITA). The limitations of these landmarks are examined, alongside variations in nerve branching patterns, mechanisms of injury, and factors related to patient characteristics and surgical experience. In addition, differences between proximal and distal approaches for nerve identification are discussed. Current strategies rely primarily on direct visualization and, in selected cases, intraoperative nerve monitoring (IONM). However, these approaches are influenced by anatomical variability and do not consistently prevent RLN injury. Available evidence suggests that no single anatomical landmark provides a universally reliable method for nerve identification. Based on these findings, we propose a surgical concept focused on identifying the RLN at its point of entry into the larynx, where the nerve typically follows a posteromedial course relative to the thyroid lobe and maintains a consistent relationship with the lateral aspect of the trachea, a region that appears more anatomically consistent and less affected by extralaryngeal variability. This approach, termed the Gamboa-Hoil principle, incorporates standard nerve identification, followed by a deliberate pause at the distal extralaryngeal segment, particularly within the final 2 cm before entry into the larynx, where the division of Berry’s ligament is deferred until the exact site of nerve entry is confirmed. This concept may provide a more consistent and reproducible framework for RLN identification. Further prospective and comparative studies are required to validate its clinical applicability and impact on surgical outcomes.

## Introduction and background

The recurrent laryngeal nerve (RLN) is a branch of the vagus nerve (cranial nerve X) and follows an indirect course through the neck [1]. The right RLN originates at the level of T1-T2 and loops around the right subclavian artery. It then ascends in a superior and medial direction toward the tracheoesophageal groove (TEG), typically positioned posterior to the thyroid lobe and lateral to the trachea, where it maintains close contact with the tracheoesophageal fascia. The left RLN arises at the level of the aortic arch, courses around it, and ascends toward the tracheoesophageal groove, remaining adjacent to this fascial plane and typically following a posteromedial orientation relative to the thyroid lobe [1,2]. Both nerves ultimately enter the larynx at approximately the C6-C7 level, typically 0.5-0.8 cm below the inferior horn of the thyroid cartilage [2,3].

In comparison, the right RLN follows a more oblique and variable course, with a sharper angulation, whereas the left RLN tends to have a longer and more vertical trajectory within the tracheoesophageal groove [1,2]. The nonrecurrent laryngeal nerve (NRLN) represents an uncommon anatomical variant, with an incidence of approximately 0.6%-1.3% on the right side and an exceptionally rare occurrence on the left (0.04%) [4]. The RLN provides motor innervation to all intrinsic laryngeal muscles and sensory innervation to the larynx below the vocal cords [1].

Thyroidectomy

Thyroid nodules are highly prevalent, affecting up to 50%-60% of the adult population, while thyroid cancer ranks among the most common malignancies worldwide, representing the seventh most frequently diagnosed cancer [5,6]. Among patients undergoing thyroidectomy, the median age was 60 years in men and 55 years in women, with a predominance of female patients (71.7% versus 28.3%) [7,8]. Indications for surgery differed slightly by sex, with malignancy accounting for 47.1% of cases in men and 40.2% in women. Total thyroidectomy was performed in 50.5% of men and 57.4% of women, whereas hemithyroidectomy was carried out in 49.6% and 42.6%, respectively [7].

RLN injury has been reported in up to 14% of thyroid surgeries, with distribution varying according to laterality [9]. Among reported injuries, approximately 41% occur on the right side, 52% on the left, and 6.3% in bilateral procedures [10]. Notably, many of these injuries are transient and do not result in significant clinical sequelae. Overall, approximately 92% of thyroidectomies are performed without major complications; however, reported adverse outcomes include hoarseness (2%), airway obstruction (1%), tracheal injury (1%), and mortality (1%) [4].

Definition of RLN injury

Vocal cord paresis was defined as the reduced mobility of the vocal cord compared to the contralateral side, whereas vocal cord paralysis was defined as the complete absence of movement. RLN injury persisting for more than 12 months was classified as permanent [9].

Clinically significant RLN paralysis has been reported in approximately 3%-5% of patients undergoing thyroid surgery. However, reported rates of RLN injury vary widely in the literature, largely reflecting differences in definitions and the inclusion of transient dysfunction, with transient injury rates ranging from 1.4% to 38% and permanent paralysis from 0.2% to 18.6%. The incidence of RLN injury is higher in patients undergoing total thyroidectomy (approximately 2%) compared to those undergoing subtotal procedures or hemithyroidectomy (approximately 0.2%) [2,9].

## Review

Search strategy and methods

A narrative literature review was conducted to evaluate the anatomical variability of the recurrent laryngeal nerve and its implications for surgical identification during thyroidectomy. A structured search of PubMed/Medical Literature Analysis and Retrieval System Online (MEDLINE), Scopus, and Web of Science was performed for articles published in English from January 1990 to January 2026, a period selected to encompass both foundational anatomical studies and contemporary surgical evidence.

Search terms included combinations of “recurrent laryngeal nerve,” “thyroidectomy,” “nerve injury,” “anatomical variation,” “Berry’s ligament,” “Zuckerkandl tubercle,” “tracheoesophageal groove,” and “inferior thyroid artery.” Articles were selected based on their relevance to surgical anatomy, mechanisms of nerve injury, and techniques for RLN identification. Reference lists of key articles and relevant reviews were also screened to identify additional studies. Study selection was performed by the author based on relevance to anatomical and surgical considerations, prioritizing studies with clear descriptions of the RLN course, variability, and intraoperative identification strategies. Given the narrative nature of this review, a formal systematic methodology and quantitative synthesis were not performed.

Inclusion and exclusion criteria

Studies were considered for inclusion if they addressed the anatomy, variability, or surgical identification of the RLN, as well as mechanisms of nerve injury during thyroidectomy. Articles describing anatomical landmarks, surgical techniques, and intraoperative considerations relevant to RLN preservation were also included. Observational studies, anatomical studies, retrospective series, and selected review articles were considered when they provided clinically relevant insights into RLN anatomy or surgical management.

Studies were excluded if they consisted of case reports, nonhuman or experimental studies without direct clinical applicability, non-English publications, or articles lacking clear relevance to RLN anatomy, variability, or intraoperative identification during thyroid surgery.

Risk-of-bias consideration

Given the narrative design of this review and the predominance of observational data, a formal risk-of-bias assessment using standardized tools was not performed. However, potential sources of bias, including selection bias, publication bias, and heterogeneity in study design, anatomical definitions, and outcome reporting, were considered during data interpretation. Particular attention was given to variability in definitions of RLN injury, differences in surgical techniques, and inconsistencies in anatomical descriptions across studies, which may influence the comparability of findings.

Safety measures

Strategies aimed at reducing the risk of RLN injury primarily rely on the careful and systematic direct visualization of the nerve during dissection, as well as the use of well-established anatomical landmarks. These approaches remain the cornerstone of safe thyroid surgery. In selected cases, intraoperative nerve monitoring (IONM) may be used as an adjunct to assist in nerve identification and functional assessment, although it does not replace meticulous surgical technique [11].

Direct visualization

The direct visualization of the RLN enables identification in the vast majority of cases (approximately 96.6%); nevertheless, complete visualization is not achieved in all patients, with reported failure rates of up to 5% [9].

Intraoperative nerve monitoring

Intraoperative nerve monitoring (IONM) is more frequently utilized in selected high-risk patients, particularly those with a history of prior neck surgery or large goiters [9]. It has been reported that up to 72.9% of patients undergoing thyroidectomy may receive neuromonitoring. Among these, transient voice changes were observed in 0.83% of cases, while 99.17% reported no alterations [5].

Loss of signal (LOS) has been reported in 3.5% of nerves at risk (NAR), with 34% of these cases associated with an abnormal nerve trajectory, suggesting a relationship between anatomical variability and the increased risk of nerve injury [12].

When combined with direct visualization, IONM has been associated with reductions in RLN injury (2.3%; p = 0.007), transient paresis (1.9%; p = 0.011), and permanent palsy (0.4%; p = 0.368) compared to visualization alone. Additionally, IONM demonstrates high specificity (96%-97%) and negative predictive value (98%-99%), supporting its reliability in confirming nerve integrity intraoperatively. However, its relatively low sensitivity (44%-63%) and limited positive predictive value (25%-38%) reduce its accuracy in predicting postoperative RLN dysfunction [13].

Permanent RLN injury remains uncommon and varies across studies; although some series report rates as low as 0.05%, these findings are influenced by differences in patient selection and surgical context. Notably, most cases of LOS have been reported in patients undergoing surgery for malignant disease (39.3%) [7].

These findings suggest that intraoperative nerve monitoring does not consistently enhance the rate of nerve identification when compared to systematic anatomical dissection. However, it appears to play a relevant role in the intraoperative recognition of complex or atypical nerve trajectories, particularly in cases of anatomical variability. These findings highlight that the primary determinant of safe RLN identification remains a precise understanding of surgical anatomy rather than reliance on adjunctive technology [5,7,9,12,13].

Berry’s ligament (BL)

In 1888, Sir James Berry described in detail the posterolateral suspensory ligament of the thyroid gland in the Journal of Anatomy of Great Britain and Ireland [14]. Berry’s ligament (BL) is a dense fibrous structure that anchors the thyroid gland to the upper tracheal rings, typically the first to third tracheal cartilages. It consists of a superficial vascular layer and a deeper, relatively avascular layer. Branches of the inferior thyroid artery (ITA) often course along its inferior margin, increasing the risk of intraoperative bleeding during dissection [3,14].

The relationship between the RLN and Berry’s ligament is highly variable. The RLN is located superficial to the ligament in approximately 78.2% of cases and deep to it in 14.8% and penetrates the ligament in about 7% of patients [3,11,15].

Notably, more than 75% of RLN injuries occur in close proximity to Berry’s ligament, often related to traction forces applied to the nerve during the medial rotation of the thyroid lobe. Intraoperative neuromonitoring studies have shown that approximately 66% of nerve injuries occur within the final 2 cm of the extralaryngeal portion of the RLN, 24% around the level of the inferior thyroid artery, and only 12% in more distal segments [14].

Berry’s ligament has traditionally been considered one of the most reliable anatomical landmarks in thyroid surgery. However, despite its structural consistency, variability in the RLN course at this level limits its reliability as a safe dissection point. Therefore, no structure in this region should be divided or ligated until the RLN has been clearly identified [11].

Importantly, the point at which the RLN enters the larynx, typically located near the cricothyroid joint, represents a more consistent anatomical reference. At this level, the nerve typically follows a posteromedial course relative to the thyroid lobe and maintains a stable relationship with the lateral aspect of the trachea. This region has been described as a safety point in thyroidectomy, offering a more reliable landmark than the variable relationship observed at the level of Berry’s ligament [14].

Zuckerkandl tubercle (ZT)

The Zuckerkandl tubercle (ZT), first described by the Austrian anatomist Emil Zuckerkandl in 1902, represents a posterolateral projection of the thyroid lobe corresponding to the fusion point between the ultimobranchial body and the median thyroid anlage. It is typically identified as a posterior extension of the lateral thyroid lobe and maintains a close anatomical relationship with the extralaryngeal terminal segment of the RLN [16].

The ZT is present in approximately 40%-90% of cases and is not identifiable in about 9.7% of patients. It is more commonly unilateral (92.9%), with a predominance on the right side (64.9%) compared to the left (35.1%), while bilateral presentation occurs in only 7.1% of cases [16].

From a surgical perspective, the RLN is most frequently located posteromedial to the ZT in approximately 80%-90% of cases; however, in up to 17% of patients, the nerve may be found in an anterior or lateral position relative to the tubercle [10,14,16].

Although the ZT can serve as a useful intraoperative guide, particularly for identifying the RLN in its terminal course, its absence in a subset of patients and the variability in nerve position limit its consistency as a surgical landmark. Therefore, it should not be relied upon as a sole reference for RLN identification [10,14,16].

Division patterns of the recurrent laryngeal nerve

The branching of the RLN is a common anatomical variation with significant surgical implications. Reported rates vary across studies, with some series describing an overall branching prevalence of approximately 10.6% [17], while others report higher rates, particularly on the right side, where divisions may occur in up to 24.1% of cases compared to 10.2% on the left [4]. Among branched nerves, bifurcation is the most frequent configuration, occurring in 90.0% of cases, whereas trifurcation (6.7%) and quadrifurcation (3.3%) are less commonly observed [17].

The mean diameter of the main RLN trunk has been reported at approximately 2.02 mm. In branched nerves, the motor branch is most commonly located in an anteromedial position (60.0%), followed by a posterolateral orientation (36.7%). In a small proportion of cases (3.3%), motor function has been identified in both branches [17].

Morphologically, motor branches tend to be significantly thicker than sensory branches (mean diameter: 1.47 mm versus 1.02 mm; p < 0.001), although both remain smaller than the main trunk. Importantly, RLN branching typically occurs at a mean distance of 2.3 cm before entering the larynx [17].

From a surgical standpoint, this variability is highly relevant, as the early division of the RLN may lead to the misidentification of a single branch as the main trunk. This is particularly critical near the terminal segment of the nerve, where inadvertent injury to a motor branch may result in clinically significant vocal cord dysfunction despite the apparent preservation of the nerve [4,17].

Inferior thyroid artery

The relationship between the RLN and the inferior thyroid artery (ITA) is variable and represents an important anatomical consideration during thyroid surgery. On the right side, approximately 68.9% of RLNs course posterior to the ITA, while 27.7% are located anterior to it. On the left side, the RLN is found posterior to the artery in 91.3% of cases and anterior in 7.3% [4].

Although the ITA may serve as an intraoperative reference, the nerve may be encountered anterior, posterior, or between its branches, limiting its consistency as a landmark for RLN identification [4].

From a clinical perspective, the identification of the RLN at the level of the ITA has not been associated with a significant reduction in nerve injury compared to identification at Berry’s ligament. Reported rates of unilateral and bilateral RLN injury were 4.4% and 2.2%, respectively, when the nerve was identified at the ITA, compared to 8.0% and 2.67% at Berry’s ligament, with no statistically significant difference (p = 0.62) [18].

Tracheoesophageal groove

The tracheoesophageal groove (TEG) is a commonly used anatomical landmark for RLN identification; however, its reliability is limited by anatomical variability. Approximately 63%-83% of RLNs are located within the groove, while up to 36% may lie outside it [10]. In a large series, the RLN was identified within the groove in 71.7% of cases and outside in 28.3%, most commonly in a lateral position (64.7%) [15].

Laterality further influences RLN position. On the right side, the RLN is located within the groove in approximately 64% of cases and often follows a more oblique and lateral course. On the left side, the RLN demonstrates a more consistent trajectory, looping around the aortic arch in 77% of cases, and may be found lateral to the trachea in 17% or anterior to it in 6% [3].

Although the TEG may serve as an initial orientation landmark, variability in nerve position, both within and outside the groove, limits its consistency as a reliable reference for RLN identification [3,10,15].

Inferior laryngeal artery

At a more distal level, the relationship between the RLN and the inferior laryngeal artery also demonstrates significant variability. On the right side, the nerve passes posterior to the artery in 50% of cases, anterior in 40%, and between arterial branches in approximately 10%. On the left side, it passes posterior to the artery in 69% of cases, anterior in 24%, and rarely between arterial branches (5%-6%) [3].

This variability further reinforces the limitations of vascular landmarks for consistent RLN identification during thyroidectomy [3].

Mechanisms and risk factors for RLN injury

RLN injury during thyroidectomy most commonly results from traction, accounting for approximately 71% of cases, and is typically associated with transient dysfunction [9-12]. Other mechanisms include direct compression or entrapment during dissection; constriction due to clips, ligatures, or fibrous bands; and thermal injury from energy-based devices such as electrocautery, harmonic scalpel, or LigaSure. Notably, thermal injury has been associated with a higher risk of permanent vocal cord paralysis, reported in up to 28% of cases. Complete transection, although the most severe form of injury, is relatively uncommon [10].

From a surgical perspective, the mechanism of injury is closely related to the approach used for nerve identification. During a lateral approach, the anatomical relationship of the RLN may be distorted, and the point at which the nerve appears closest to the midline does not necessarily correspond to its true entry into the larynx. Instead, this point may represent a more peripheral segment of the nerve, frequently at the level of Berry’s ligament [14].

This observation is clinically relevant, as it highlights the potential risk of misidentifying the RLN when relying on lateral dissection alone, particularly in regions of increased anatomical variability and higher susceptibility to traction-related injury [14].

Age

Age has also been identified as a relevant factor associated with increased risk of complications following thyroidectomy. Elderly patients (≥65 years) demonstrate a higher incidence of overall RLN injury compared to younger individuals (2.3% versus 1.0%; OR, 1.58; 95% CI, 1.15-2.16; I² = 76%). Additionally, increased rates of postoperative hematoma (2.4% versus 1.0%; OR, 2.32; 95% CI, 1.70-3.16; I² = 0%) and mortality during follow-up (0.3% versus 0.01%; OR, 11.09; 95% CI, 1.77-69.52; I² = 90%) have been reported in this population [19].

These findings suggest that patient-related factors, such as age, may further increase vulnerability to RLN injury, particularly in the context of anatomically complex dissections [19].

Surgeon experience and volume

Surgeon experience and procedural volume have been consistently associated with postoperative outcomes in thyroid surgery. Surgeons performing more than 100 thyroidectomies per year have been reported to achieve significantly lower complication rates (approximately 4.3%), whereas those performing fewer than 10 procedures annually may experience more than a fourfold increase in complication rates [20].

These findings highlight the importance of surgical expertise and case volume as key determinants of patient safety, particularly in procedures involving critical structures such as the RLN [20].

Taken together, these findings indicate that RLN injury is a multifactorial event influenced by anatomical variability, surgical technique, and patient-related factors, reinforcing the need for a consistent and reliable method of nerve identification [14,19,20].

Proximal versus distal RLN identification

Different surgical approaches have been described for RLN identification, including proximal and distal techniques. In a cohort of 209 patients (59 undergoing proximal identification and 150 distal identification), both groups were comparable in baseline characteristics [21].

Despite the proximal approach being more frequently employed in complex cases, characterized by a higher rate of lateral dissection (42.4% versus 26.0%; p = 0.037) and a greater number of lymph nodes resected (13.28 ± 6.65 versus 9.55 ± 5.93; p < 0.001), no significant differences were observed in long-term RLN function (p = 1.000). Additionally, no differences were found in postoperative calcium supplementation (p = 0.644) [21].

These findings suggest that both proximal and distal approaches are safe and yield comparable functional outcomes, even in more technically demanding scenarios [21].

However, these results also indicate that the surgical approach itself may not be the primary determinant of RLN preservation. Instead, the accurate identification of the nerve at a consistent and reliable anatomical point may play a more critical role in preventing injury [21].

Surgical concept proposal: The Gamboa-Hoil principle

The evidence presented in this review highlights a fundamental limitation in current strategies for RLN identification. Commonly used anatomical landmarks, including Berry’s ligament, the Zuckerkandl tubercle, the tracheoesophageal groove, and the inferior thyroid artery, demonstrate significant variability and do not consistently reduce the risk of nerve injury. Additionally, variations in branching patterns, surgical approach, and patient- and surgeon-related factors further complicate reliable RLN identification.

In this context, we propose a surgical concept based on the identification of the RLN at its most anatomically constant location: its point of entry into the larynx. At this level, the nerve is typically located in a posteromedial position relative to the thyroid lobe and enters the larynx in close proximity to the cricothyroid joint, maintaining a consistent relationship with the lateral aspect of the trachea.

The Gamboa-Hoil principle can be summarized in three key components: (1) the initial identification and standard dissection of the RLN along its course; (2) the recognition of the distal extralaryngeal segment, particularly within the final 2 cm before laryngeal entry, as a high-risk zone where the nerve lies in close posteromedial relation to the thyroid lobe and is frequently adherent to Berry’s ligament (in this region, dissection should be paused and the division of Berry’s ligament deferred); and (3) the precise identification of the nerve at its laryngeal entry point prior to completing the division of the superior portion of Berry’s ligament to ensure the safe mobilization of the thyroid gland.

**Figure 1:**
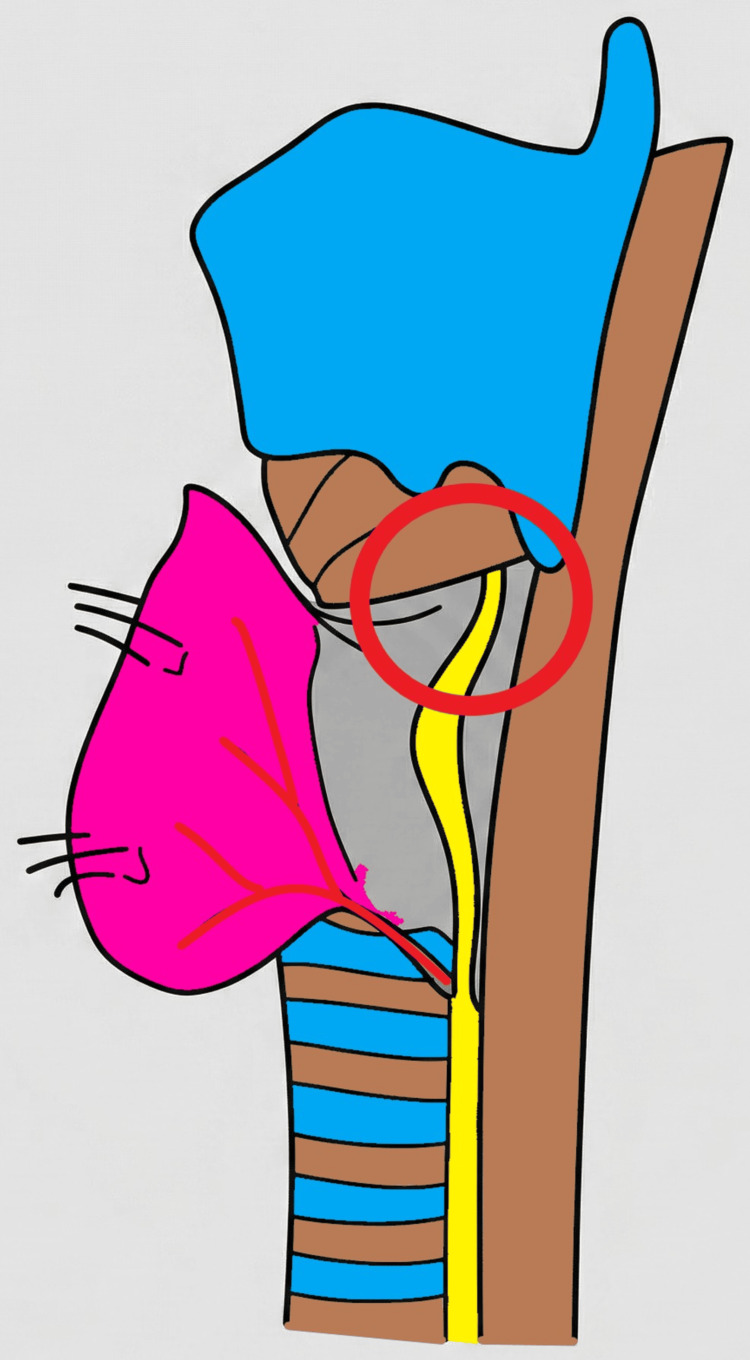
Schematic representation of the Gamboa-Hoil principle for recurrent laryngeal nerve (RLN) identification during thyroidectomy. The circled area highlights the distal extralaryngeal segment of the RLN, particularly within the final 2 cm before entry into the larynx, which represents a high-risk zone for nerve injury. At this level, the nerve typically lies in a posteromedial position relative to the thyroid lobe and is frequently adherent to Berry’s ligament. The concept emphasizes a deliberate pause in dissection and the deferral of Berry’s ligament division until the nerve is precisely identified at its laryngeal entry point. Image credit: original illustration created by the author.

This approach introduces a deliberate pause at the most vulnerable segment of the nerve, constituting a “stop-before-risk zone” strategy (Figure 1).

By establishing the laryngeal entry point as the primary anatomical reference, this concept may reduce the risk of misidentification associated with proximal or lateral approaches and help prevent inadvertent injury to terminal branches, particularly in cases of early nerve division. From a technical perspective, the principle does not replace conventional dissection but refines it by incorporating a targeted pause and confirmation step at a critical anatomical segment before completing ligament division. This may be particularly advantageous in anatomically complex cases, including those with distorted anatomy, large goiters, malignancy, or prior surgery.

From a clinical perspective, surgical experience and procedural volume have been consistently associated with improved outcomes in thyroid surgery, with high-volume surgeons demonstrating lower complication rates, including RLN injury. However, achieving such proficiency requires substantial operative exposure, which may not be feasible in all clinical or training settings. In this context, the Gamboa-Hoil principle may offer a structured and anatomically consistent framework for RLN identification that is applicable across different levels of surgical experience.

Importantly, this proposed concept does not replace traditional anatomical knowledge or established surgical principles but rather integrates them into a more consistent framework for nerve identification. By prioritizing the most reliable anatomical reference point, the Gamboa-Hoil principle aims to enhance surgical safety and reduce the incidence of RLN injury.

Further prospective and comparative studies are required to validate this approach and to determine its impact on clinical outcomes; however, the anatomical and surgical rationale presented supports its potential role as a standardized strategy in thyroid surgery.

Limitations

This study has several limitations that should be acknowledged. First, as a narrative review, this study does not follow a systematic methodology, which may introduce selection bias and limit the reproducibility of the search and study selection process. Although a structured search strategy was employed, the absence of a formal systematic framework restricts the ability to ensure the full replicability of the results.

Second, the findings presented are based on heterogeneous studies with variations in study design, definitions of RLN injury, and surgical techniques. This heterogeneity may affect the comparability of reported outcomes and limits the ability to draw definitive conclusions.

Third, the Gamboa-Hoil principle is proposed based on anatomical rationale and the interpretation of existing evidence rather than direct prospective clinical validation. Although the concept incorporates a structured intraoperative strategy, particularly a deliberate pause at the distal extralaryngeal segment, a region characterized by a close posteromedial relationship of the nerve to the thyroid lobe and increased adherence to Berry’s ligament, its impact on reducing RLN injury rates has not yet been evaluated in controlled clinical studies.

Finally, factors such as surgeon experience, institutional volume, and case complexity may influence outcomes and were not uniformly accounted for across the reviewed literature. These variables may affect the generalizability of the proposed concept.

Future prospective and comparative studies are required to validate the clinical applicability, safety, and reproducibility of this approach in different surgical settings.

## Conclusions

RLN injury remains a critical concern in thyroid surgery, primarily due to the considerable anatomical variability of the nerve and its inconsistent relationship with traditional surgical landmarks. Although direct visualization and intraoperative nerve monitoring contribute to safer dissection, neither approach fully overcomes the limitations imposed by anatomical variation. The evidence reviewed demonstrates that commonly used landmarks, including Berry’s ligament, the Zuckerkandl tubercle, the tracheoesophageal groove, and the inferior thyroid artery, do not provide a universally reliable method for RLN identification. In addition, factors such as nerve branching patterns, surgical approach, patient characteristics, and surgeon experience further influence the risk of injury, underscoring the multifactorial nature of RLN damage.

In this context, identifying the RLN at its point of entry into the larynx emerges as a more consistent and anatomically reliable strategy. At this level, the nerve typically follows a posteromedial course relative to the thyroid lobe and maintains a consistent relationship with the lateral aspect of the trachea. The Gamboa-Hoil principle, based on standard nerve identification, followed by a deliberate pause at the distal extralaryngeal segment, particularly within the final 2 cm before laryngeal entry, prior to the division of Berry’s ligament, offers a structured framework that may improve the safety and reproducibility of RLN identification during thyroidectomy. Importantly, this concept is applicable across different levels of surgical experience and may serve as a useful adjunct to established surgical principles rather than a replacement. Further prospective and comparative studies are warranted to validate its clinical impact; however, the anatomical and surgical rationale support its potential role as a standardized approach in thyroid surgery.
